# The *Geobacillus* Pan-Genome: Implications for the Evolution of the Genus

**DOI:** 10.3389/fmicb.2016.00723

**Published:** 2016-05-24

**Authors:** Oliver K. Bezuidt, Rian Pierneef, Amin M. Gomri, Fiyin Adesioye, Thulani P. Makhalanyane, Karima Kharroub, Don A. Cowan

**Affiliations:** ^1^Department of Genetics, Centre for Microbial Ecology and Genomics, University of PretoriaPretoria, South Africa; ^2^Department of Biochemistry, Centre for Bioinformatics and Computational Biology, University of PretoriaPretoria, South Africa; ^3^Equipe Métabolites des Extrêmophiles, Laboratoire de Recherche Biotechnologie et Qualité des Aliments, Institut de la Nutrition de l’Alimentation et des Technologies Agro-Alimentaire, Université des Frères MentouriConstantine, Algeria

**Keywords:** *Geobacillus*, pan-genome, horizontal gene transfer, conserved core, flexible genomes, soft core

## Abstract

The genus *Geobacillus* is comprised of a diverse group of spore-forming Gram-positive thermophilic bacterial species and is well known for both its ecological diversity and as a source of novel thermostable enzymes. Although the mechanisms underlying the thermophilicity of the organism and the thermostability of its macromolecules are reasonably well understood, relatively little is known of the evolutionary mechanisms, which underlie the structural and functional properties of members of this genus. In this study, we have compared 29 *Geobacillus* genomes, with a specific focus on the elements, which comprise the conserved core and flexible genomes. Based on comparisons of conserved core and flexible genomes, we present evidence of habitat delineation with specific *Geobacillus* genomes linked to specific niches. Our analysis revealed that *Geobacillus* and *Anoxybacillus* share a high proportion of genes. Moreover, the results strongly suggest that horizontal gene transfer is a major factor deriving the evolution of *Geobacillus* from *Bacillus*, with genetic contributions from other phylogenetically distant taxa.

## Introduction

The genus *Geobacillus* comprises a diverse group of Gram-positive aerobic and facultative anaerobic endospore-forming bacterial species. Based on 16S rRNA gene sequence similarity these bacteria were all classified as a separate unit designated as group 5 of thermophilic *Bacillus* (Ash et al., 1991). As the genus *Bacillus* was previously reported to be genetically extremely heterogeneous (Ash et al., 1991), its taxonomy was revised and the species assigned to group 5 were reclassified as members of the genus *Geobacillus*, with *Geobacillus stearothermophilus* (Donk, 1920; Nazina et al., 2001) as the type strain based on DNA–DNA hybridization, fatty acid and 16SrRNA gene analyses (Nazina et al., 2001). The *Geobacillus* strains, which have been sequenced and reported to date show an average genome size of 3.5–3.9 Mbp and a G + C content ranging from 45 to 55% (Hussein et al., 2015). These genomes include *G. thermoleovorans*, *G. kaustophilus*, *G. thermocatenulatus*, *G. thermodenitrificans*, *G. stearothermophilus*, *G. caloxylosilyticus* and *G. thermoglucosidasius*, which are members of the 15 validly reported *Geobacillus* species (Brumm et al., 2015) form 9 distinct sequence similarity groups based on phylogenies constructed with 16S rRNA and *rec*N genes (Nazina et al., 2001; Zeigler, 2005). Four distinct phylogenetic clusters are formed using the variant single-nucleotide sites of their core genome (Studholme, 2015).

Members of the genus *Geobacillus* are facultative thermophiles, growing at optimum temperatures ranging from 45 to 75°C (Coorevits et al., 2012). They are ubiquitous in natural and man-made thermal environments, including hydrothermal pools, desert soils, waste-treatment plants, hot water pipelines, dairy-processing, and mining environments and compost (Marchant et al., 2002; Kimura et al., 2003; Deflaun et al., 2007; Zhao et al., 2012; Bezuidt et al., 2015) and may also be isolated from a variety of non-thermal sites (Corwin, 2002). *Geobacillus* species are important in the field of biotechnology due to their diverse degradative and biosynthetic physiologies (Cripps et al., 2009; De Maayer et al., 2014; Hussein et al., 2015; Studholme, 2015) and for the production of multiple thermostable enzymes (Shariff et al., 2011; Bhalla et al., 2015).

Ecological diversity in bacteria is typically a result of micro-evolutionary events, such as horizontal gene transfer (HGT), which are tightly linked with microbial adaptation and evolution (Boto, 2010). While the evolutionary relationships and major traits of *Bacillus* species have been previously explored (Alcaraz et al., 2010), these interactions have not been specifically defined in *Geobacillus*. To understand the effect of HGT in shaping the evolution of *Geobacillus* from *Bacillus*, we apply comparative genomics approaches, focusing on the core, soft-core, shell, and cloud genomes.

## Materials and Methods

### Genome Sequences

The 29 *Geobacillus* genomes used for pan-genome analyses as well as the 19 *Bacillus* and 13 *Anoxybacillus* genomes, used for gene conservancy analyses were acquired from the NCBI^1^. The characteristics of all strains are summarized in the Supplementary Table S1.

### Pan-Genome Analysis and Clustering

The methodology for creating orthologous gene clusters was previously described by Contreras-Moreira and Vinuesa (2013). Briefly, to allocate genes into the different categorical orthologous levels, GET_HOMOLOGUES (Contreras-Moreira and Vinuesa, 2013) was used to conduct sequence similarity searches and clustering of the coding sequences (CDSs) from the 29 genomes using pair-wise BLASTP (Altschul et al., 1990) and OrthoMCL (OMCL; Li et al., 2003) algorithms. For the identification and clustering of genes into different orthologous groups the parameters were set as: -*E* < 1e-05 expectation value for blastp searches; -*C* > 75% minimum alignment coverage to qualify sequences as best hits; -*t* 0 reporting all the computed clusters and; -*F* 1.5 OMCL inflation parameter. The four clusters determined from the analyses were defined as previously described (Koonin and Wolf, 2008; Kaas et al., 2012): core – genes present in all the genomes; softcore – genes present in 95% (≥28) of the genomes; shell – genes present in >3 and <26 of the genomes; cloud – genes present in <2 of the genomes.

### Average Amino Acid Identities amongst *Geobacillus* Homologous CDSs

A GET_HOMOLOGUES script was used to estimate the average amino acid identities of CDSs between individual members of a pan-genome. The percent identities of protein coding genes in the 4 clusters in the 29 genomes were determined in the form of a Gower’s distance matrix using a script from GET_HOMOLOGUES. The distance matrices were further illustrated in the form of a heatmap to show similarities and differences between genomes.

### Functional Classification of Orthologous Genes

The four clusters determined for the 29 genomes were searched for pattern similarity using a standalone RPS-BLAST (reverse position specific blast) with -*E*<1e-05 against a conserved domain database of clusters of orthologous groups (COG; Tatusov et al., 2000)^2^. Genes with pattern similarities were assigned functional classes, which were later categorized into different COG subgroups to determine their distributions for all the cluster compartments.

### Identification of Carbohydrate Active Enzymes

The dbCAN database (Yin et al., 2012) was used to search the clusters for the presence of different families of carbohydrate-active enzymes (CAZymes; Alalouf et al., 2011) and their associated domains. Each cluster was searched for pattern similarity using hmmscan (Eddy, 1998) against the CAZymes family specific hidden markov model (HMM; Rabiner, 1989). The results obtained were processed to determine the abundances and distributions of the different CAZymes families and their domains within each cluster.

### Introgression of Genomic Regions between Divergent Populations

All elements contained within the four clusters were compared against the Predicted Genomic Islands database (Pre_GI; Pierneef et al., 2015) by means of BLASTP using a cutoff *E* value <1e-05. Pre_GI is a collection of horizontally acquired genetic material identified in 2,407 bacterial/archaeal organisms and entails 656,806 proteins from diverse sources. The highest scoring hit for each element in a cluster was determined and all four clusters were individually analyzed with respect to host taxonomy, host general information, and CDS description of the subject. The majority of sequences, in all four clusters, that displayed no significant similarity were described as “hypothetical proteins” and excluded from further analysis.

### Homology Searches of the *Geobacillus* Pan-Genome in *Anoxybacillus* and *Bacillus* Genomes

A large-scale Blast score ratio (LS-BSR; Sahl et al., 2014) was used to determine the variable composition of genes in each pan-genome cluster within the 13 *Anoxybacillus* and 19 *Bacillus* genomes to infer their evolution and phylogeny. The TBLASTN BSR values calculated for the genomes against the clusters were converted into matrices of 1’s and 0’s. Here, genes with BSR ≥ 0.8 were considered to be conserved (1) and those with BSR < 0.8 were designated as divergent (0) between the clusters and genomes. The matrices were displayed as heatmaps to show similarities and differences between the genomes and clusters.

### Average Nucleotide Identities amongst *Geobacillus* and *Anoxybacillus* CDSs

The GET_HOMOLOGUES methodology was used to compare 29 *Geobacillus* and 13 *Anoxybacillus* genomes to estimate average nucleotide identities of their CDSs by means of BLASTN. The percent identities of the CDSs between the genomes were determined in the form of a Gower’s distance matrix using a GET_HOMOLOGUES functionality. The distance matrix was visualized as a heatmap to show similarities and differences between genomes.

### Geo_Island Prediction and Homology

All *Geobacillus* strains/isolates were subjected to the SeqWord Gene Island Sniffer (SWGIS; Bezuidt et al., 2009) for island prediction. SWGIS is a standalone island predictor employing oligonucleotide usage (OU) frequencies to isolate areas of horizontal transfer in archaeal and bacterial genomes. OU frequencies establish microbial genomic signatures and local deviations from the global pattern indicate regions of probable horizontal transfer. OU pattern (OUP) using 4-mer frequencies, embodied in an island allows for the determination of compositional similarity between islands by correlating lists of consecutively similar word patterns. Sequence comparison among islands was obtained by BLASTN analysis. To increase the reliability of possible homology between genomic islands, we combined data derived from compositional and sequence-based comparison methods (Pierneef et al., 2015).

## Results and Discussion

While members of the genus *Geobacillus* are well known for their ecological, physiological and genetic diversity (Zeigler, 2014; Studholme, 2015), it is unclear how their diverse environments shape genomic composition and how this may in turn influence their lifestyles. We conducted pan-genomic analysis on the 29 genomes (12 complete and 17 draft genomes from NCBI) derived from *Geobacillus* isolates of geographically distinct origins.

**FIGURE 1:**
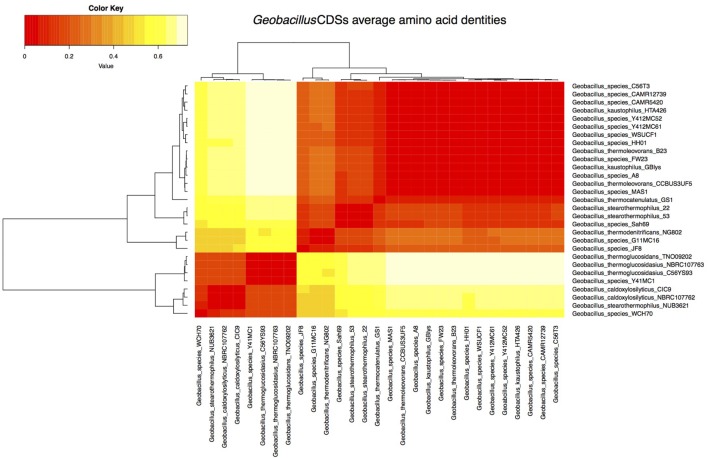
**Heatmap representing the degree of similarity of the genomes based on the average amino acid identities of their protein coding genes.** The heatmap was derived from an average amino acid identity matrix determined from the high similarity (dark orange) and low similarity (light yellow) of CDSs in the 4 pan-genomic clusters derived from the 29 *Geobacillus* genomes.

The full complement of genes in the pan-genome included 13,595 clusters of protein-coding genes. Among these 527, 1,862, 3,515, and 8,218 clusters represented the core, soft-core, shell, and cloud genomes, respectively, (**Supplementary Figure S1**). The ‘core genome’ represents a pool of conserved genes, which are present in all genomes included in the analysis. The ‘soft-core’ represents genes present in 95% of the genomes analyzed. The inclusion of this category is important in comparative genomic analyses as it allows for the inclusion of draft genomes where some genes may not be present (Nelson and Stegen, 2015). Both the core and soft-core clusters represent a pool of highly conserved genes, which can provide information about the evolutionary history of members of the genus (Nelson and Stegen, 2015). The ‘shell’ cluster includes genes, which are moderately common in the pan-genome (i.e., 3 to 26 genomes of the 29 genomes included in these analyses). The ‘cloud’ cluster represents genes which are present in very few of the genomes analyzed (2 or less). Both the shell and cloud clusters represent subsets of the flexible genome, which reflect both the evolutionary history of a sublineage and the lifestyle and adaptation of an organism to its particular environment (Nelson and Stegen, 2015). These two clusters are thought to have different rates of gene acquisition and deletion (Collins and Higgs, 2012). The shell is believed to include genes that are gained and lost rather slowly, whereas the cloud is comprised of genes that are rapidly gained and lost (Collins and Higgs, 2012). From pan-genomic analyses, average amino acid identity matrices were calculated using protein-CDSs within the clusters to compare and classify the 29 genomes (Supplementary Table S2). The comparisons are shown in the form of a heatmap (**Figure 1**), which depicts the clustering of genomes into five groups based on average shared similarities and differences of their CDSs amino acid identities (core and flexible gene pools combined) relative to the four determined by Studholme (2015). The latter illustrates the degree of HGT in microbial evolution and also displays a functional relationship between different *Geobacillus* strains obtained from variable environments.

The functional annotation of the four clusters was performed using stand-alone rps-blast against the COG PSSMs from the CDD database. The distributions of the COG categories were determined by counting all the individual subcategories assigned to genes of each cluster compartment. 435/527 (83%) core, 1571/1862 (84%) soft-core, 2150/3515 (61%) shell, and 2883/8218 (35%) cloud genes were assigned to the COG categories. The assignments were subsequently used to determine the fraction of the individual compartment genes in each of the different COG functional categories (**Supplementary Figure S2**). The majority of the COG categories were overrepresented in the flexible genome relative to the conserved core: these included genes involved in replication, recombination and repair (L), amino acid transport and metabolism (E), carbohydrate transport and metabolism (G), transcription (K), energy production and conversion (C), signal transduction mechanisms (T), defense mechanisms (V), and secondary metabolites biosynthesis, transport, and catabolism (**Supplementary Figure S2**). The conserved core was overrepresented by genes in the COG category (J) of translation and ribosomal structure genes and partially overrepresented by categories coenzyme transport and metabolism (H), nucleotide transport and metabolism (F), protein turnover and chaperones genes (O), as seen in *Bacillus* (Alcaraz et al., 2010). Only one COG category (N), cell motility, was found to have a similar distribution of genes between the conserved core and flexible genomes (Alcaraz et al., 2010). The overrepresentation of the COG categories in the flexible genome (rather than the core genome) is thought to be the principal driver of *Geobacillus* functional diversity. These results suggest that HGT may be a key mechanism of the adaptive nature of *Geobacillus*.

The dbCAN database was used to annotate and determine the distribution and associations of the different CAZymes within the four clusters. The dbCAN analysis provides HMM profiles derived from protein coding genes, which contain CAZyme domains classified into five enzymatic classes: glycosyl transferases (GTs), glycoside hydrolases (GHs), polysaccharide lyases (PLs), carbohydrate esterases (CEs) and auxiliary activities (Kaas et al., 2012), and (non-enzymatic) carbohydrate-binding modules (CBMs). The distributions of the CAZyme types were determined by counting all the individual classes assigned to genes of each cluster compartment. The majority of the CAZyme genes and domains of classes GHs, GTs, and CBM were overrepresented in the flexible gene pool relative to the core genome (Supplementary Table S3). The annotations for the clusters were also compared to CAZymes previously reported for the 16 *Geobacillus* strains and cataloged in the CAZy database (Supplementary Table S4). Of the different CAZyme classes identified within the four clusters the following: GH 74–113 and 127, GT 7, 12–13, 70–71 and 94, CE 1–3, AA 2–4 and 7, CBM 16, 23, 37–40, 51–56 and 66–67, and PL 9 families were found to be absent from the CAZyme families previously reported for *Geobacillus* and were also overrepresented in the flexible genome (Supplementary Table S4). The overrepresentation of these classes in the flexible genome, relative to the core genome, highlights the importance of HGT and its contribution to the diversity in the metabolic machinery of *Geobacillus* species and consequently, to their ecological importance and biotechnological potential.

**FIGURE 2:**
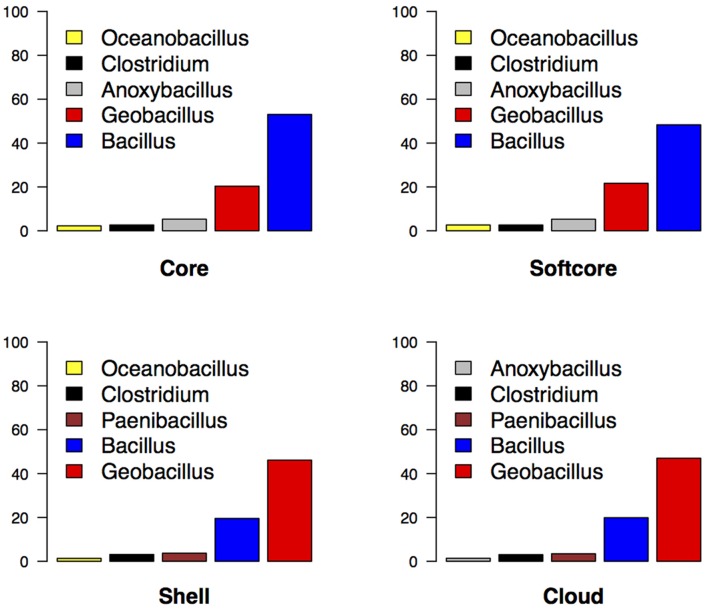
**Bar plots representing the frequency of the top five genera with regards to sequence similarity of proteins of the core, softcore, shell, and cloud against proteins contained in Pre_GI.** Only the highest scoring hit subject for a protein was included to avoid over-representation of certain genera.

Protein sequences of the four clusters were compared to the Pre_GI database by means of BLASTP. High-scoring alignments were inspected with regard to the genus and general information on the organism with which sequence similarity was identified. The core cluster of *Geobacillus* displayed a strong homology to *Bacillus*, with a progressive change in overrepresentation to that of *Geobacillus* in subsequent clusters (**Figure 2**). This indicates the presence of a *Bacillus* ancestry in both the conserved core clusters, with the flexible genome clusters highly influenced by *Geobacillus*. The mechanistic implications are that the ancestral *Bacillus* genome has acquired, by HGT, a wide variety of bacterial and archaeal genes, the acquisition of which has led to the evolution of the genus *Geobacillus*. Such acquisitive processes are also likely to have led to the development of ‘extremophilic’ physiology of *Geobacillus*, including varying degrees of thermo- and halophilicity (Aravind et al., 1998; Koonin and Wolf, 2008). The core cluster also displayed a moderate representation of *Anoxybacillus*, a genus that has been reported to be a closet phylogenetic neighbor to *Geobacillus* and to share a high gene synteny with both *Geobacillus* and *Bacillus* (Saw et al., 2008).

**FIGURE 3:**
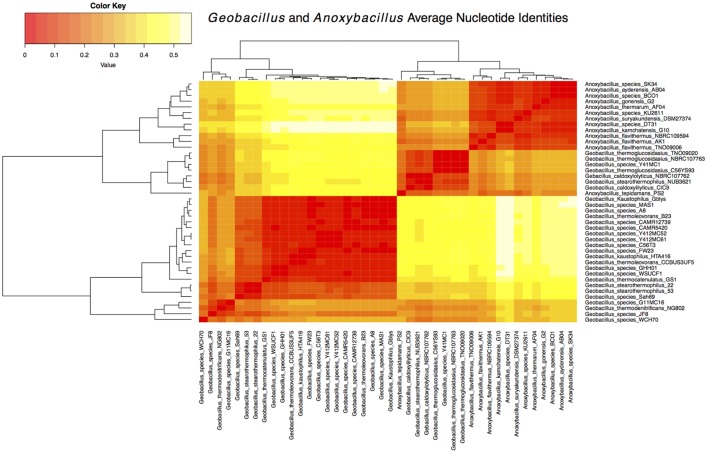
**Heatmap representing the degree of similarity among *Geobacillus* and *Anoxybacillus* based on the average nucleotide identities of their coding sequences (CDSs).** The heatmap was derived from an average nucleotide identity matrix determined from the high similarity (dark orange) and low similarity (light yellow) of CDSs derived from the *Geobacillus* and *Anoxybacillus* genomes.

The protein sequences from each cluster were further compared with the complete and draft genomes of *Bacillus* and *Anoxybacillus*, using LS-BSR to determine the difference and proportion of genes shared within the three genera. The BSR matrix values (Supplementary Table S5) were used to review the gene conservancy average for each cluster against *Bacillus* and *Anoxybacillus* before visualization using MultiExperiment viewer (MeV version 4.9; **Supplementary Figures S3–S6**). The four clusters revealed high a degree of sequence similarity and gene composition, mainly for the core clusters shared between *Geobacillus* and *Anoxybacillus*. The gene conservancy average for *Anoxybacillus* were found to be: core 527 (171.8), soft-core 1862 (554.8), shell 3515 (187.9), cloud 8218 (397.1). Similarly, the values for *Bacillus* were found to be: core 527 (75.8), soft-core 1862 (216.1), shell 3515 (39.6), and cloud 8218 (84.5). Of the 13 *Anoxybacillus* genomes compared with the clusters, *Anoxybacillus tepidamans* PS2 [formally known as *G. tepidamans* (Minana-Galbis et al., 2010)] displayed the highest abundance genes from the core 527 (230), soft-core 1862 (759), and the shell 3515 (309) clusters. *A. thermarum* harboured the second highest abundance of genes from the cloud 8218 (485). As *Geobacillus* and *Anoxybacillus* were shown to be closely related, their genomes were compared to determine the average nucleotide identity matrices for their CDSs (Supplementary Table S6) and how similar these are among the genera. The matrices were illustrated as a heatmap, which depicts *A. tepidamans* PS2 clustering with *Geobacillus* whereas the other 12 *Anoxybacillus* clustering on their own (**Figure 3**).

**FIGURE 4:**
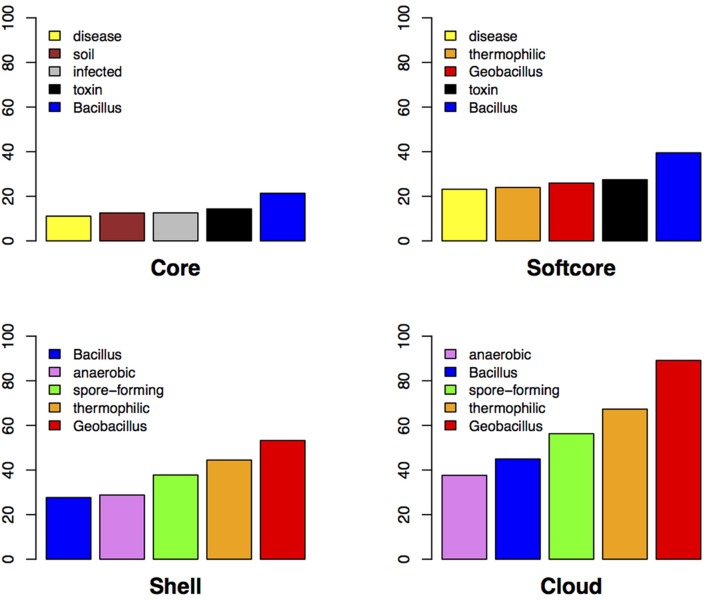
**Graphical representation of general information regarding the genera of highest scoring hits against Pre_GI with only the highest subject included.** The top five word frequencies reflecting island host lifestyle, habitat and isolation are presented in the bar plot for the core, softcore, shell, and cloud.

The possible influence of environmental factors on the different cluster levels is shown in **Figure 4**. Our analysis reveals that genes, which contribute to organismal adaptation to challenging environmental conditions, are typically found in the flexible genome clusters. Environmental pressures and adaptation to niche environments is thought to have played a critical role in the evolution of *Geobacillus* from *Bacillus* (Alalouf et al., 2011). To further understand the role of HGT in the evolution of *Geobacillus*, all 29 genomes were inspected for the presence of genomic islands using SWGIS (Pierneef et al., 2015). The analysis identified 567 regions (geo_islands). Elements of the core, softcore, shell, and cloud were aligned to the geo_islands to identify the presence/absence of proteins in a horizontally acquired region. From the core genome, 357 of the 527 proteins were represented in a geo_island (67.74%) with the softcore accommodating 1,341 of the possible 1,862 (72.02%) proteins. The shell contained the highest proportion of proteins in a geo_island with 2,915 out of 3,515 (82.93%), while the cloud contained 5,835 proteins from the set of 8,218 (71.00%). The high proportion of genetic elements in the core, softcore, shell, and cloud, which are located in probable regions of horizontal transfer, may indicate the extent and influence of HGT on all categories of the *Geobacillus* genome.

All geo_islands were compared individually against Pre_GI by means of sequence (BLASTN) and compositional (tetranucleotide frequency) analyses. Sequence similarity indicated that only 2.12% of the top hits were not homologous to an island predicted in a strain of *Geobacillus*, while compositional similarity analysis showed that only 1.24% of geo_islands were possibly not of *Geobacillus* origin. This highlights the high frequency of HGT within the genus *Geobacillus.* These geo_islands serve as a genetic reservoir for members of *Geobacillus* when environmental changes are encountered and rapid evolution is crucial in order to survive.

## Conclusion

This work provides the first insights on the importance of HGT toward the evolution of *Geobacillus*. Based on the full complement of genes determined from the 29 *Geobacillus* genomes, we were able to distinguish and define the functional roles of genes present within both the core and flexible genomes and how these contribute toward diversification of the genus. The results obtained from the COG and CAZymes analyses, suggested that the majority of genes and enzymes implicated in adaptation were overrepresented in the flexible rather than the core genome. Further sequence-based analyses on the core and flexible genomes, matched against the Pre_GI datasets, indicated that the core genome was similar to that of *Bacillus*, whereas the flexible genome shows similarities shared within *Geobacillus* (and other organisms) as a result of multiple HGT events. Similarities shared between the core genome and the *Bacillus* Pre_GI genomic islands; suggest that *Bacillus* may have contributed toward the evolution of *Geobacillus*. For further exploration, these clusters were compared with the complete and draft genomes of *Bacillus* and *Anoxybacillus* species. The *Geobacillus* core (predominantly) and flexible genomes revealed a high level of similarity with *Anoxybacillus*, which may indicate a recent divergence of the two genera. Further comparative genomics analyses is still required in order to infer the phylogenetic relationships of the three genera, which would shed light on the influence of *Bacillus* on the evolutionary processes of both *Geobacillus* and *Anoxybacillus*. Furthermore, our analysis suggest that *A. tepidamans* PS2 should still be regarded a *Geobacillus* based on their shared genes and ANI properties.

## Author Contributions

OB, RP, and FA conducted the bioinformatic analysis and wrote the first draft of the paper, AG isolated one of the *Geobacillus* isolates used in this study, TM, KK, and DC conceived the study and provided reagents. All authors contributed to writing the manuscript.

## Conflict of Interest Statement

The authors declare that the research was conducted in the absence of any commercial or financial relationships that could be construed as a potential conflict of interest.

## Footnotes

1 ftp://ftp.ncbi.nih.gov/genomes/genbank/bacteria/

2 ftp://ftp.ncbi.nlm.nih.gov/pub/mmdb/cdd/little_endian/

## Supplementary Material

**FIGURE S1 | Barplot of pan-genome matrix determined from the *Geobacillus* genomes.**

**FIGURE S2 | Clusters of Orthologous Groups (COG) analysis of the *Geobacillus* pan-genome**.

**FIGURE S3 | Heatmap comparison of the Geobacillus core genes against Anoxybacillus and Bacillus genomes**.

**FIGURE S4 | Heatmap comparison of the Geobacillus soft-core genes against Anoxybacillus and Bacillus genomes**.

**FIGURE S5 | Heatmap comparison of the Geobacillus shell genes against Anoxybacillus and Bacillus genomes**.

**FIGURE S6 | Heatmap comparison of the Geobacillus cloud genes against Anoxybacillus and Bacillus genomes**.

## References

[B1] Alalouf O. Balazs Y. Volkinshtein M. Grimpel Y. Shoham G. Shoham Y. (2011). A new family of carbohydrate esterases is represented by a GDSL hydrolase/acetylxylan esterase from *Geobacillus stearothermophilus*. *J. Biol. Chem.* 286 41993–42001. 10.1074/jbc.M111.301051 21994937 PMC3234920

[B2] Alcaraz L. D. Moreno-Hagelsieb G. Eguiarte L. E. Souza V. Herrera-Estrella L. Olmedo G. (2010). Understanding the evolutionary relationships and major traits of *Bacillus* through comparative genomics. *BMC Genomics* 11:332 10.1186/1471-2164-11-332 PMC2890564 20504335

[B3] Altschul S. F. Gish W. Miller W. Myers E. W. Lipman D. J. (1990). Basic local alignment search tool. *J. Mol. Biol.* 215 403–410. 10.1016/S0022-2836(05)80360-2 2231712

[B4] Aravind L. Tatusov R. L. Wolf Y. I. Walker D. R. Koonin E. V. (1998). Evidence for massive gene exchange between archaeal and bacterial hyperthermophiles. *Trends Genet.* 14 442–444. 10.1016/S0168-9525(98)01553-4 9825671

[B5] Ash C. Farrow J. A. E. Wallbanks S. Collins M. D. (1991). Phylogenetic heterogeneity of the genus *Bacillus* revealed by comparative analysis of small-subunit-ribosomal RNA sequences. *Lett. Appl. Microbiol.* 13 202–206. 10.1111/j.1472-765X.1991.tb00608.x

[B6] Bezuidt O. Lima-Mendez G. Reva O. N. (2009). SeqWord Gene Island Sniffer: a program to study the lateral genetic exchange among bacteria. *World Acad. Sci. Eng. Technol.* 58 1169–1174.

[B7] Bezuidt O. K. Makhalanyane T. P. Gomri M. A. Kharroub K. Cowan D. A. (2015). Draft genome sequence of thermophilic *Geobacillus* sp. Strain Sah69, isolated from Saharan Soil, Southeast Algeria. *Genome Announc.* 3:e1447–15 10.1128/genomeA.01447-15 PMC4683223 26679578

[B8] Bhalla A. Bischoff K. M. Sani R. K. (2015). Highly thermostable xylanase production from A thermophilic *Geobacillus* sp. Strain WSUCF1 utilizing lignocellulosic biomass. *Front. Bioeng. Biotechnol.* 3:84 10.3389/fbioe.2015.00084 PMC4468944 26137456

[B9] Boto L. (2010). Horizontal gene transfer in evolution: facts and challenges. *Proc. Biol. Sci.* 277 819–827. 10.1098/rspb.2009.1679 19864285 PMC2842723

[B10] Brumm P. Land M. L. Hauser L. J. Jeffries C. D. Chang Y.-J. Mead D. A. (2015). Complete genome sequences of *Geobacillus* sp. Y412MC52, a xylan-degrading strain isolated from obsidian hot spring in Yellowstone National Park. *Stand. Genomic Sci.* 10:81 10.1186/s40793-015-0075-0 PMC4617443 26500717

[B11] Collins R. E. Higgs P. G. (2012). Testing the infinitely many genes model for the evolution of the bacterial core genome and pangenome. *Mol. Biol. Evol.* 29 3413–3425. 10.1093/molbev/mss163 22752048

[B12] Contreras-Moreira B. Vinuesa P. (2013). GET_HOMOLOGUES, a versatile software package for scalable and robust microbial pangenome analysis. *Appl. Environ. Microbiol.* 79 7696–7701. 10.1128/AEM.02411-13 24096415 PMC3837814

[B13] Coorevits A. Dinsdale A. E. Halket G. Lebbe L. De Vos P. Van Landschoot A. (2012). Taxonomic revision of the genus *Geobacillus*: emendation of *Geobacillus*, *G. stearothermophilus*, *G. jurassicus*, *G. toebii*, *G. thermodenitrificans* and *G. thermoglucosidans* (nom. corrig., formerly ‘thermoglucosidasius’); transfer of *Bacillus thermantarcticus* to the genus as *G. thermantarcticus* comb. nov.; proposal of *Caldibacillus debilis* gen. nov., comb. nov.; transfer of *G. tepidamans* to *Anoxybacillus* as *A. tepidamans* comb. nov.; and proposal of *Anoxybacillus* caldiproteolyticus sp. nov. *Int. J. Syst. Evol. Microbiol.* 62 1470–1485. 10.1099/ijs.0.030346-0 21856988

[B14] Corwin P. (2002). What are high-temperature bacteria doing in cold environments? *Trends Microbiol.* 10 120–121. 10.1016/S0966-842X(02)02311-9 11864820

[B15] Cripps R. E. Eley K. Leak D. J. Rudd B. Taylor M. Todd M. (2009). Metabolic engineering of Geo*bacillus* thermoglucosidasius for high yield ethanol production. *Metab. Eng.* 11 398–408. 10.1016/j.ymben.2009.08.005 19703579

[B16] De Maayer P. Brumm P. J. Mead D. A. Cowan D. A. (2014). Comparative analysis of the *Geobacillus* hemicellulose utilization locus reveals a highly variable target for improved hemicellulolysis. *BMC Genomics* 15:836 10.1186/1471-2164-15-836 PMC4194401 25273399

[B17] Deflaun M. F. Fredrickson J. K. Dong H. Pfiffner S. M. Onstott T. C. Balkwill D. L. (2007). Isolation and characterization of a *Geobacillus* thermoleovorans strain from an ultra-deep South African gold mine. *Syst. Appl. Microbiol.* 30 152–164. 10.1016/j.syapm.2006.04.003 16709445

[B18] Donk P. J. (1920). A highly resistant thermophilic organism. *J. Bacteriol.* 5 373–374. 16558885 10.1128/jb.5.4.373-374.1920 PMC378892

[B19] Eddy S. R. (1998). Profile hidden markov models. *Bioinformatics* 14 755–763. 10.1093/bioinformatics/14.9.755 9918945

[B20] Hussein A. H. Lisowska B. K. Leak D. J. (2015). The genus *Geobacillus* and their biotechnological potential. *Adv. Appl. Microbiol.* 92 1–48. 10.1016/bs.aambs.2015.03.001 26003932

[B21] Kaas R. S. Friis C. Ussery D. W. Aarestrup F. M. (2012). Estimating variation within the genes and inferring the phylogeny of 186 sequenced diverse *Escherichia coli* genomes. *BMC Genomics* 13:577 10.1186/1471-2164-13-577 PMC3575317 23114024

[B22] Kimura H. Asada R. Masta A. Naganuma T. (2003). Distribution of microorganisms in the subsurface of the manus basin hydrothermal vent field in Papua New Guinea. *Appl. Environ. Microbiol.* 69 644–648. 10.1128/AEM.69.1.644-648.2003 12514053 PMC152445

[B23] Koonin E. V. Wolf Y. I. (2008). Genomics of bacteria and archaea: the emerging dynamic view of the prokaryotic world. *Nucleic Acids Res.* 36 6688–6719. 10.1093/nar/gkn668 18948295 PMC2588523

[B24] Li L. Stoeckert C. J. Jr. Roos D. S. (2003). OrthoMCL: identification of ortholog groups for eukaryotic genomes. *Genome Res.* 13 2178–2189. 10.1101/gr.1224503 12952885 PMC403725

[B25] Marchant R. Banat I. M. Rahman T. J. Berzano M. (2002). The frequency and characteristics of highly thermophilic bacteria in cool soil environments. *Environ. Microbiol.* 4 595–602. 10.1046/j.1462-2920.2002.00344.x 12366754

[B26] Minana-Galbis D. Pinzon D. L. Loren J. G. Manresa A. Oliart-Ros R. M. (2010). Reclassification of *Geobacillus pallidus* (Scholz et al. 1988) Banat et al. 2004 as Aeri*bacillus pallidus* gen. nov., comb. nov. *Int. J. Syst. Evol. Microbiol.* 60 1600–1604. 10.1099/ijs.0.003699-0 19700455

[B27] Nazina T. N. Tourova T. P. Poltaraus A. B. Novikova E. V. Grigoryan A. A. Ivanova A. E. (2001). Taxonomic study of aerobic thermophilic bacilli: descriptions of *Geobacillus subterraneus* gen. nov., sp. nov. and *Geobacillus uzenensis* sp. nov. from petroleum reservoirs and transfer of *Bacillus* stearothermophilus, *Bacillus thermocatenulatus*, *Bacillus thermoleovorans*, *Bacillus kaustophilus*, *Bacillus thermodenitrificans* to *Geobacillus* as the new combinations *G. stearothermophilus*, G. th. *Int. J. Syst. Evol. Microbiol.* 51 433–446. 11321089 10.1099/00207713-51-2-433

[B28] Nelson W. C. Stegen J. C. (2015). The reduced genomes of *Parcubacteria* (OD1) contain signatures of a symbiotic lifestyle. *Front. Microbiol.* 6:713 10.3389/fmicb.2015.00713 PMC4508563 26257709

[B29] Pierneef R. Bezuidt O. Reva O. N. (2015). Optimization and practical use of composition based approaches towards identification and collection of genomic islands and their ontology in prokaryotes. *Procedia Comput. Sci.* 51 670–679. 10.1016/j.procs.2015.05.183

[B30] Rabiner L. R. (1989). A tutorial on hidden markov-models and selected applications in speech recognition. *Proc. IEEE* 77 257–286. 10.1109/5.18626

[B31] Sahl J. W. Caporaso J. G. Rasko D. A. Keim P. (2014). The large-scale blast score ratio (LS-BSR) pipeline: a method to rapidly compare genetic content between bacterial genomes. *PeerJ* 2:e332 10.7717/peerj.332 PMC3976120 24749011

[B32] Saw J. H. Mountain B. W. Feng L. Omelchenko M. V. Hou S. Saito J. A. (2008). Encapsulated in silica: genome, proteome and physiology of the thermophilic bacterium *Anoxybacillus flavithermus* WK1. *Genome Biol.* 9:R161 10.1186/gb-2008-9-11-r161 PMC2614493 19014707

[B33] Shariff F. M. Rahman R. N. Basri M. Salleh A. B. (2011). A newly isolated thermostable lipase from *Bacillus* sp. *Int. J. Mol. Sci.* 12 2917–2934. 10.3390/ijms12052917 21686158 PMC3116164

[B34] Studholme D. J. (2015). Some (bacilli) like it hot: genomics of *Geobacillus* species. *Microb. Biotechnol.* 8 40–48. 10.1111/1751-7915.12161 25195706 PMC4321371

[B35] Tatusov R. L. Galperin M. Y. Natale D. A. Koonin E. V. (2000). The COG database: a tool for genome-scale analysis of protein functions and evolution. *Nucleic Acids Res.* 28 33–36. 10.1093/nar/28.1.33 10592175 PMC102395

[B36] Yin Y. B. Mao X. Z. Yang J. C. Chen X. Mao F. L. Xu Y. (2012). dbCAN: a web resource for automated carbohydrate-active enzyme annotation. *Nucleic Acids Res.* 40 W445–W451. 10.1093/nar/gks479 22645317 PMC3394287

[B37] Zeigler D. R. (2005). Application of a recN sequence similarity analysis to the identification of species within the bacterial genus *Geobacillus*. *Int. J. Syst. Evol. Microbiol.* 55 1171–1179. 10.1099/ijs.0.63452-0 15879251

[B38] Zeigler D. R. (2014). The *Geobacillus* paradox: why is a thermophilic bacterial genus so prevalent on a mesophilic planet? *Microbiology* 160 1–11. 10.1099/mic.0.071696-0 24085838

[B39] Zhao Y. Caspers M. P. Abee T. Siezen R. J. Kort R. (2012). Complete genome sequence of *Geobacillus thermoglucosidans* TNO-09.020, a thermophilic sporeformer associated with a dairy-processing environment. *J. Bacteriol.* 194 4118 10.1128/JB.00318-12 PMC3416512 22815439

