# Supplementary figures and images for: The Geobacillus Pan-Genome: Implications for the Evolution of the Genus
Source: Front Microbiol. 2016 May 24;7:723. doi: 10.3389/fmicb.2016.00723 (PMC4878294; doi:10.3389/fmicb.2016.00723)

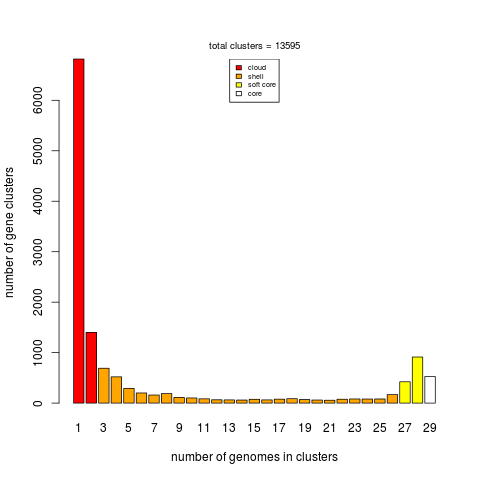

Supplement: Supplementary file 1 [file Image_1.PNG]

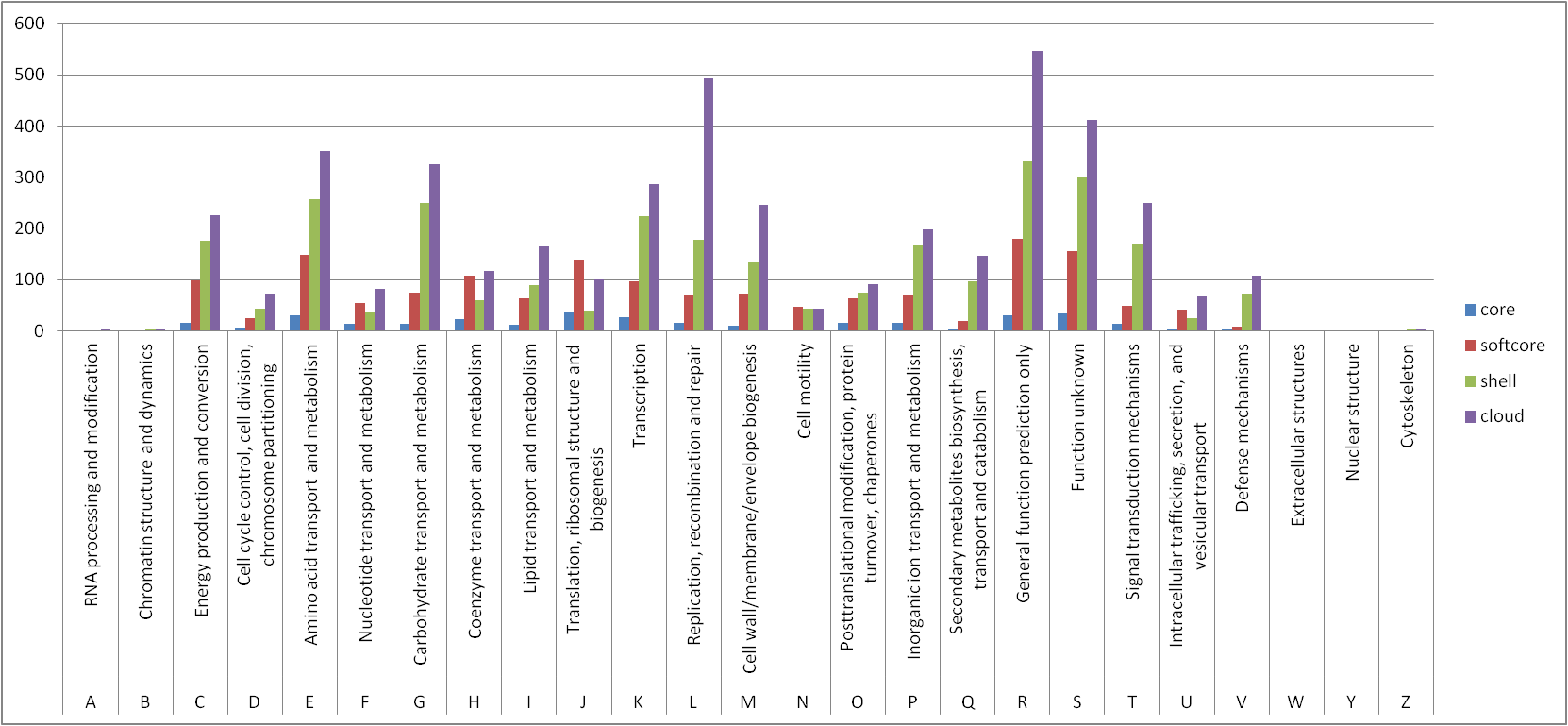

Supplement: Supplementary file 2 [file Image_2.PNG]

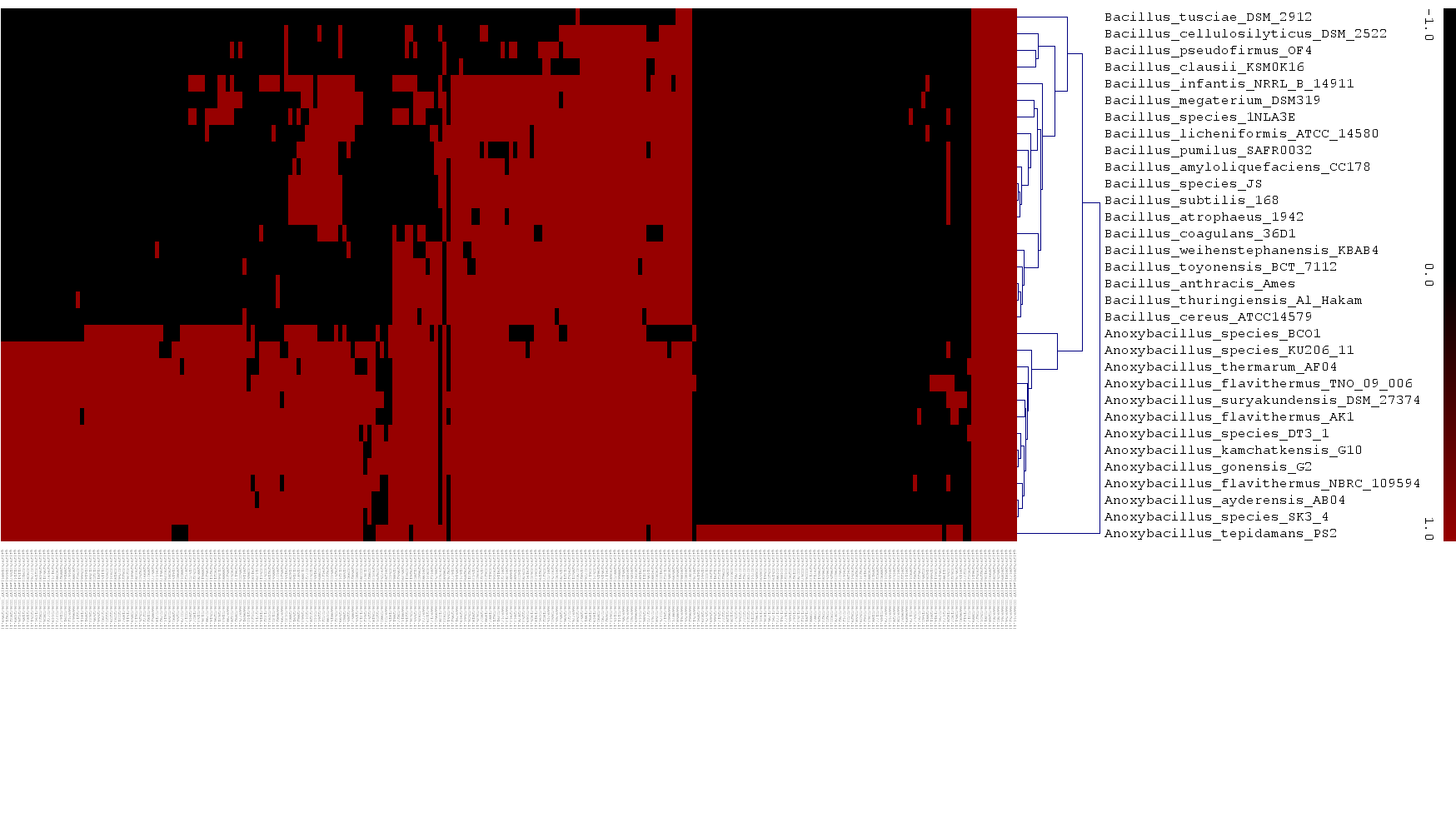

Supplement: Supplementary file 3 [file Image_3.PNG]

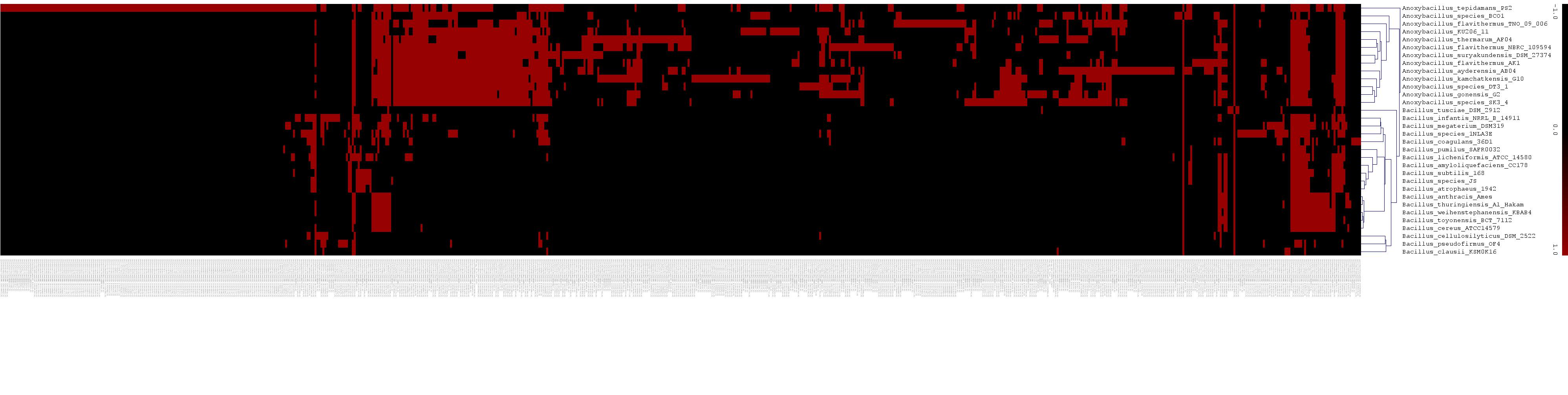

Supplement: Supplementary file 4 [file Image_4.PNG]

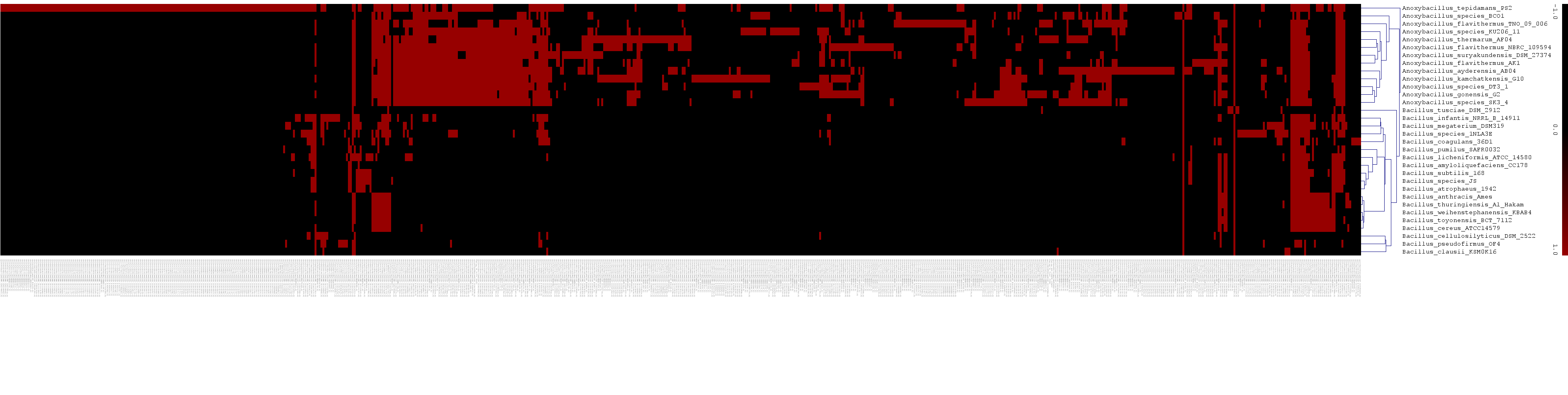

Supplement: Supplementary file 5 [file Image_5.PNG]

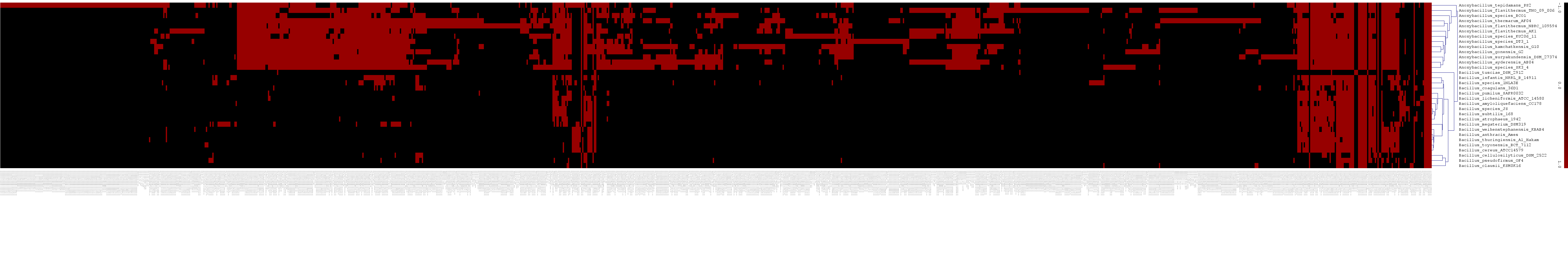

Supplement: Supplementary file 6 [file Image_6.PNG]
